# Genotype–phenotype correlations in 294 pediatric patients with osteogenesis imperfecta

**DOI:** 10.1093/jbmrpl/ziae125

**Published:** 2024-09-30

**Authors:** Jay J Byrd, Andrew C White, Claire G Nissen, Makayla Schissel, Matthew Van Ormer, Danita Velasco, Maegen Wallace

**Affiliations:** College of Medicine, University of Nebraska Medical Center, Omaha, NE, 68198, United States; The Child Health Research Institute, University of Nebraska Medical Center, Omaha, NE, 68198, United States; College of Medicine, University of Nebraska Medical Center, Omaha, NE, 68198, United States; College of Medicine, University of Nebraska Medical Center, Omaha, NE, 68198, United States; Department of Biostatistics, College of Public Health, University of Nebraska Medical Center, Omaha, NE, 68198, United States; The Child Health Research Institute, University of Nebraska Medical Center, Omaha, NE, 68198, United States; Children’s Nebraska, Department of Pediatrics, Division of Genetics Omaha, NE, 68114, United States; Phoenix Childrens Hospital, Department of Pediatric Orthopaedic Surgery Phoenix, AZ, 85016, United States

**Keywords:** genotype–phenotype, OI, pediatrics, orthopedics, genetic variants

## Abstract

Osteogenesis imperfecta (OI) is an inherited disorder characterized by bone fragility with extraskeletal manifestations mostly due to *COL1A1* and *COL1A2* variants. Currently, 23 genes have been implicated in the pathogenesis of OI; however, literature on genotype–phenotype correlation and incidence of non-skeletal clinical features are limited. This study aims to identify genotype–phenotype correlations in patients with OI, allowing clinicians to better inform families of prognosis, optimize patient care, and facilitate evidence-based clinical decision-making. We retrospectively reviewed 294 patients with OI to collect demographic data, clinical characteristics, and genotypic information. Patients were stratified by *COL1A1/1A2* vs non-*COL1A1/1A2* variants to evaluate differences in phenotype. The majority of OI was due to variants in *COL1A1/1A2* (91%), with the remaining 9% due to non-*COL1A1/1A2* variants. Most patients in the *COL1A1/2* group were White compared to the non-*COL1A1/2* group (78% vs 50%; *p* = 0.004). *COL1A/1A*2 patients had higher incidence of blue sclerae (83% vs 58%, *p* = 0.002), dentinogenesis imperfecta (49% vs 15%, *p* < 0.001), and family history of OI (34% vs 12%, *p* = 0.03). Those in the non-*COL1A1/1A2* group have higher rates of scoliosis compared to those in the *COL1A1/1A2* group (62% vs 40%, *p* = 0.04), as well as higher rates of expressive language disorder/delay (15% vs 0.4% in non-*COL1A1/1A2* and *COL1A1/1A2* patients, respectively; *p* < 0.001). Identifying the underlying molecular etiology early is imperative for optimal clinical care, allowing for appropriate risk counseling, identification of affected relatives, and improved anticipatory care and management. These data support that rare subtypes of OI occur more frequently in non-White individuals and demonstrated genetic associations with incidence of blue sclera, dentinogenesis imperfecta, scoliosis, and expressive language disorders.

## Introduction

Osteogenesis imperfecta (OI) is a heterogenous connective tissue disorder with an estimated incidence of 1 per 10 000 individuals.^1^ The core phenotype of OI is bone fragility with recurrent fractures, skeletal deformity, and short stature; however, multiple secondary features have been described. OI is caused by dominant, recessive, or X linked variants in multiple genes resulting in variable phenotypes affecting both skeletal and extraskeletal tissues.^1^ The majority of OI is due to autosomal dominant variants in *COL1A1* or *COL1A2,* the genes that code for the alpha chains of type I collagen, the most abundant protein in the bone matrix which is integral to its structure. People with *COL1A1* and *COL1A2* variants typically either have a quantitative changes resulting in decreased amounts of normal type I collagen, mainly the result of frameshift or nonsense mutations.^2^ Alternatively, those with more severe forms of the disease tend to have qualitative changes, typically the result amino acid substitutions in polypeptide chains, resulting in structurally abnormal type I collagen.^3^

Autosomal dominant variants in these genes were first associated with OI in the late 1970s.^4^ Classification of OI types I-IV was proposed by Sillence et al. in 1979 based on clinical and radiographic features and inheritance patterns, which has been the standard since that time. Types II and III are the most severe, with type II typically resulting in perinatal demise, followed in severity by types IV and I. Individuals with type I typically have grossly normal stature but remain prone to frequent fractures.^5^ In 2015, a revision to the Nosology and Classification of Genetic Skeletal Disorders distinguished 5 OI types based on clinical presentation.^6^ As bioinformatics and sequencing technology has grown in use and accessibility, it has catalyzed the discovery of new autosomal recessive variants in *non-COL1A1/A2* genes in OI, necessitating the Genetic classification of 18 types 1 yr later in 2016.^7^ The most recent nosology of genetic skeletal disorders published in 2023 has grouped skeletal disorders with one of the groups the OI and bone fragility group. The current recommendation for naming OI is to note the phenotypic severity and what gene is related to the disorder.^8^ Currently, the Online Mendelian Inheritance in Man (MIM) database lists new types of OI with the discovery of novel genes (https://www.ncbi.nlm.nih.gov/omim/).

Of non-*COL1A1/A2* variants, genetic defects leading to compromised bone mineralization, defects in collagen post-translational modification, aberrant collagen processing and cross-linking, or altered osteoblast differentiation and function have been discovered in patients with OI.^1^ While this new information improves patient care and genetic counseling, the phenotypic and clinical heterogeneity of those with non-*COL1A1/1A2* variants often complicate classification and prognosis. Additionally, the large number of candidate genes further complicates the identification of consistent genotype–phenotype presentations.

Many have studied genotype–phenotype correlations in OI^2^^,^^9–18^; however, these were conducted with relatively small sample sizes at institutions outside the United States and typically have focused on those with *COL1A1/1A2* inherited defects. The present study aims to augment current literature with one of the largest cohorts evaluated and further elucidate the complex relationships and phenotypic differences between those with *COL1A1/1A2* and *non-COL1A1/1A2* variants.

## Materials and methods

### Study population

We retrospectively reviewed all patients with a diagnosis of OI (ICD code Q78.0) who were treated at Children’s Nebraska in Omaha, Nebraska between January 1990 and June 2022. A total of 447 patients were initially identified from the electronic health record and our institution’s OI Database; however, 107 patients were excluded as they did not have a clinical OI type documented. An additional 46 patients were excluded due to incomplete medical records resulting in a total of 294 patients included in the final analysis. Approval for the study was obtained from the University of Nebraska Medical Center Institutional Review Board (#0498-22-EP).

Patient demographics (sex, age in months, ethnicity, clinical OI type, and genetic variant) were collected for each patient. Clinical characteristics were collected and include the presence of blue sclera, dentinogenesis imperfecta (DI), hearing loss- and age at first hearing loss if applicable, bone mineral density, use of bisphosphonates, rod implantation for long bone deformity, scoliosis- and scoliosis operations if applicable, family history of OI, joint hypermobility, and if the patient has been diagnosed with short stature. This study also evaluated the prevalence of non-OI diagnoses including the prevalence of autism spectrum disorder (ASD), expressive language disorders/delays, seizures, tics, and hydrocephalus or necessity of ventriculoperitoneal shunt (VPS). Assessments occurred with annual multidisciplinary clinic evaluations including long bone and spinal radiographs, DEXA scans, physical and occupational therapy evaluations, and routine pulmonary and audiologic evaluation.

Demographics and clinical characteristics were compared for patients with *COL1A1/1A2* variants to patients with non-*COL1A1/1A2* variants. Patients were further stratified using a functional metabolic classification proposed by Forlino and Marini. This consists of 5 functional groups: group A consists of primary defects in collagen structure/processing (*COL1A1*, *COL1A2*, and *BMP1*), group B is due to defects in collagen modification (*CRTAP*, *LEPRE1*, *PPIB*, and *TMEM38B*), group C relates to defects in collagen folding and cross-linking (*SERPINH1*, *FKBP10*, and *PLOD2*), group D consists of defection in ossification/mineralization (*IFITM5* and *SERPINF1*), and group E with defects in osteoblast development with collagen insufficiency (*WNT1*, *CREB3L1*, and *SP7*).^18^

### Statistical analysis

Descriptive statistics which included means and standard deviations for all continuous variables and counts and percentages for categorical responses were used to summarize the data. Three new continuous variables were created: age at first hearing loss diagnosis, age at first bisphosphonate infusion, and age at DEXA scan. Age values were rounded down to the nearest whole integer. Chi square or Fisher’s Exact test were used as appropriate to compare all categorical variables by *COL* groups. Independent samples t-tests or Wilcoxon Rank Sum tests were used to compare continuous variables by *COL* groups. All analyses were conducted using SAS version 9.4. *p* < 0.05 was considered statistically significant.

## Results

### Study population

Of the 294 individuals included in this study, the majority had *COL1A1/1A2* variants (*n* = 268, 91.2%). There was equal representation between males and females, overall, and no significant difference in gender representation between groups (*p* = 0.22). There was a significant association between race and *COL1A1/1A2* grouping. The majority of patients in the *COL1A1/1A2* group were White compared to the non-*COL1A1/1A2* group (78% vs 50%; *p* = 0.002). Population characteristics are displayed stratified by *COL* vs non-*COL* grouping and by individual grouping in Tables 1 and 2, respectively.

**Table 1:** Raw data and statistics stratified by *COL* vs non-*COL* mutation.

	***COL* groups**			
	***COL*** (*N* = 268)	**Non-*COL*** (N = 26)	**Total** (*N* = 294)	***p*-value**
**Sex, *n* (%)**				0.22^a^
** Female**	131 (48.9%)	16 (61.5%)	147 (50.0%)	
** Male**	137 (51.1%)	10 (38.5%)	147 (50.0%)	
**Race, *n* (%)**				**0.004** ^b^
** White**	209 (78.0%)	13 (50.0%)	222 (75.5%)	
** Non-White**	59 (22.0%)	13 (50.0%)	72 (24.5%)	
**Autism, *n* (%)**	4 (1.5%)	2 (7.7%)	6 (2.0%)	0.09^b^
**Expressive language disorder/delay, *n* (%)**	1 (0.4%)	4 (15.4%)	5 (1.7%)	**0.0002** ^b^
**Blue sclera, *n* (%)**	221 (82.5%)	15 (57.7%)	236 (80.3%)	**0.002** ^a^
**Dentinogenesis imperfecta, *n* (%)**	131 (48.9%)	4 (15.4%)	135 (45.9%)	**0.0009** ^b^
**Age at first hearing loss diagnosis, mean (SD)**	8.6 (6.16)	3.8 (2.99)	8.2 (6.09)	0.13^c^
**Age at first bisphosphonate infusion, mean (SD)**	3.9 (4.52)	2.5 (3.36)	3.7 (4.44)	0.39^c^
**Age at DEXA, mean (SD)**	11.4 (5.10)	8.9 (4.59)	11.2 (5.10)	**0.02** ^d^
**Patient received bisphosphonates at time of DEXA, *n* (%)**	231 (87.5%)	26 (100.0%)	257 (88.6%)	0.05^b^
**DEXA Z-score, mean (SD)**	−1.1 (1.73)	−0.9 (2.08)	−1.1 (1.76)	0.68^d^
**Family history of OI, *n* (%)**	91 (34.0%)	3 (11.5%)	94 (32.0%)	**0.03** ^b^
**History of scoliosis, *n* (%)**	108 (40.4%)	16 (61.5%)	124 (42.3%)	**0.04** ^a^
**Scoliosis operations, *n* (%)**	21 (19.4%)	1 (6.3%)	22 (17.7%)	0.30^b^
**History of rod placement for long bone deformity, *n* (%)**	184 (68.7%)	20 (76.9%)	204 (69.4%)	0.38^a^
**Presence of joint hypermobility, *n* (%)**	207 (77.2%)	21 (80.8%)	228 (77.6%)	0.68^a^
**Patient diagnosed short in stature, *n* (%)**	145 (54.1%)	19 (73.1%)	164 (55.8%)	0.06^a^

^a^Chi-square *p*-value;

^b^Fisher Exact *p*-value;

^c^Wilcoxon rank sum *p*-value;

^d^Equal variance 2 sample t-test.

Abbreviations: DEXA, Dual-Energy X-ray Absorptiometry; SD, standard deviation. Bolded values are the statistically significant values.

**Table 2:** Descriptive statistics of outcomes by individual grouping.

	**Grouping by metabolic defect**				
	**Group A** (*N* = 268)	**Group B** (*N* = 11)	**Group C** (N = 1)	**Group D** (*N* = 10)	**Group E** (*N* = 4)
**Sex, *n* (%)**					
** Female**	131 (48.9%)	6 (54.5%)	1 (100.0%)	6 (60.0%)	3 (75.0%)
** Male**	137 (51.1%)	5 (45.5%)	0 (0.0%)	4 (40.0%)	1 (25.0%)
**Race, *n* (%)**					
** White**	209 (78.0%)	4 (36.4%)	0 (0.0%)	7 (70.0%)	2 (50.0%)
** Non-White**	59 (22.0%)	7 (63.6)	1 (100.%)	2 (20.0%)	13 (50.0%)
**Autism, *n* (%)**	4 (1.5%)	1 (9.1%)	0 (0.0%)	0 (0.0%)	1 (25.0%)
**Expressive language disorder/delay, *n* (%)**	1 (0.4%)	3 (27.3%)	0 (0.0%)	1 (10.0%)	0 (0.0%)
**Blue sclera, *n* (%)**	221 (82.5%)	7 (63.6%)	1 (100.0%)	5 (50.0%)	2 (50.0%)
**Dentinogenesis imperfecta, *n* (%)**	131 (48.9%)	2 (18.2%)	0 (0.0%)	2 (20.0%)	0 (0.0%)
**Age at first hearing loss diagnosis, mean (SD)**	8.6 (6.16)	2.0 (1.41)		3.0 (..)	8.0 (.)
**Age at first bisphosphonate infusion, mean (SD)**	3.9 (4.52)	2.5 (4.61)	3.0 (.)	2.0 (1.76)	3.8 (3.30)
**Age at DEXA, mean (SD)**	11.4 (5.10)	7.0 (4.84)	8.0 (.)	10.0 (4.16)	11.5 (4.36)
**Patient received bisphosphonates at time of DEXA, *n* (%)**	231 (87.5%)	11 (100.0%)	1 (100.0%)	10 (100.0%)	4 (100.0%)
**DEXA Z-score, mean (SD)**	−1.1 (1.73)	−1.7 (2.21)	−3.0 (.)	0.4 (1.63)	−1.8 (1.41)
**Family history of OI, *n* (%)**	91 (34.0%)	0 (0.0%)	0 (0.0%)	3 (30.0%)	0 (0.0%)
**History of scoliosis, *n* (%)**	108 (40.4%)	7 (63.6%)	1 (100.0%)	7 (70.0%)	1 (25.0%)
**Scoliosis operations, *n* (%)**	21 (19.4%)	0 (0.0%)	0 (0.0%)	1 (14.3%)	0 (0.0%)
**History of rod placement for long bone deformity, *n* (%)**	184 (68.7%)	9 (81.8%)	1 (100.0%)	7 (70.0%)	3 (75.0%)
**Presence of joint hypermobility, *n* (%)**	207 (77.2%)	9 (81.8%)	0 (0.0%)	8 (80.0%)	4 (100.0%)
**Patient diagnosed short in stature, *n* (%)**	145 (54.1%)	8 (72.7%)	0 (0.0%)	7 (70.0%)	4 (100.0%)

Descriptive statistics only for the comparison of Groups A-E due to small sample sizes.

Abbreviations: DEXA, Dual-Energy X-ray Absorptiometry; SD, standard deviation. The values were intentionally left blank as the *N* was too small for a SD.

### OI genotype–phenotype relationships

Blue sclerae were significantly associated with genotype, being present in 83% and 58% of those with *COL1A1/1A2* and *non-COL1A1/A2* variants, respectively (*p* = 0.002). DI was also more common in the *COL1A1/1A2* vs the non-*COL1A1/A2* group (49% vs 15%, *p* < 0.001).

Similar proportions of enrolled patients in both the *COL1A1/1A2* and the n*on-COL1A1/1A2* experienced hearing loss (15.7% vs 15.4%, respectively, *p* = 1.00). Participants in the *COL1A1/1A2* group were more likely to be diagnosed with hearing loss later into childhood than participants in the non-*COL1A1/1A2* group, but the difference was not significant (*p* = 0.13). Hearing loss was first diagnosed in the *COL1A1/1A2* group at a median age of 7.0 (IQR: 3.0-14.0) while the non-*COL1A1/1A2* group had a median diagnosis age of 3.0 (IQR: 2.0-5.5) yr old.

Most of the enrolled study participants have received bisphosphonate infusions (96.6%) and DEXA scans (99.0%). Both the *COL1A1/1A2* and non-*COL1A1/1A2* groups had high rates of bisphosphonate infusions (96.2% vs 100%, respectively). The median age at which the participant received their first bisphosphonate infusion did not significantly differ between the *COL1A1/1A2* and non-*COL1A1/1A2* groups (2.0 yr old [IQR: 0.0-6.0] vs 2.0 yr old [IQR: 0.0-3.0, *p* = 0.39]). The proportion of participants who had a DEXA scan was similar between the 2 groups, with 98.9% of *COL1A1/1A2* and 100% of the non-*COL1A1/1A2* participants. There was a significant difference in the average age of participants at their first DEXA scan between the *COL1A1/1A2* and non-*COL1A1/1A2* groups (11.4 yr [SD: 5.10] vs 8.9 yr [SD: 4.59, *p* = .02]). The results of the DEXA scans were not statistically significant (*p* = 0.68) between the 2 groups, with the mean Z-score for the *COL1A1/1A2* group of –1.1 (SD: 1.73) and –0.9 (SD: 2.08) for the non-*COL1A1/1A2* group. There was a moderate association in the proportion of patients receiving bisphosphonate infusions at the time of DEXA between the *COL1A1/1A2* and non-*COL1A1/1A2* groups (87.5% vs 100%, *p* = 0.05).

Nearly 70% of all patients had a history of rod placement for long bone deformity. Additionally, 42.3% of the participants in this study have scoliosis. Those in the non-*COL1A1/1A2* group have significantly higher rates of scoliosis compared to those in the *COL1A1/1A2* group (61.5% vs 40.4%, *p* = 0.04). However, it appears that the *COL1A1/1A2* group has higher rates of scoliosis operations compared to the non-*COL1A1/1A2* group, though not significant (19.4% vs 6.3%, *p* = 0.30).

The majority of all patients (55.8%) had a diagnosis of short stature; this was not significantly different between the groups (54.1% vs 73.1%, *p* = 0.06). Approximately 78% of all patients had joint hypermobility present, with no significant difference between the *COL1A1/1A2* and non-*COL1A1/1A2* groups (77.2% vs 80.8%, *p* = 0.68). Lastly, 34% of those in the *COL1A1/1A2* group have a family history of OI, compared to only 12% of those in the non-*COL1A1/1A2* group (*p* = 0.03).

### Non-OI genotype phenotype relationships

Expressive language disorder/delay was significantly associated with *COL1A1/1A2* grouping, with a prevalence of 15% and 0.4% in non-*COL1A1/1A2* and *COL1A1/1A2* patients, respectively (*p* < 0.001). ASD also had a higher, but insignificant, prevalence in non-COL groups compared to COL groups, though not significant (7.7% vs 1.5% respectively, *p* = 0.09). The presence of tics, seizures, and hydrocephalus is low across both groups (*p* = 1.00 for all 3 criteria). Tics were not reported in either grouping. Only one seizure was reported in the *COL1A1/1A2* group. There were 3 reports of VPS in the *COL1A1/1A2* group.

## Discussion

The present study shows varying prevalence of certain phenotypic characteristics in OI when comparing individuals with *COL1A1/1A2* to those with non*-COL1A1/1A2* variants. To the best of our knowledge, this study utilizes one of the largest OI cohorts studied in the United States.

The presence or absence of blue sclera is a commonly studied finding of interest in those examining phenotypic differences in OI. We found that those with *COL1A1/1A2* variants were significantly more likely (*p* = 0.002) to have blue sclera than those with non-*COL1A1/1A2* variants. Similar findings have been reported in the prevalence of blue sclera but have often looked at different parameters. For instance, a study by Liu et al. in 2017 found that those with autosomal dominant inheritance, which are typically *COL1A1/1A2* variants, were more likely to have blue sclera than those with autosomal recessive inheritance, typically of non-*COL1A1/1A2* genes.^13^ Furthermore, several studies have shown that individuals with more mild clinical forms of the disease (OI type I), are significantly more likely to present with blue sclera than those with more severe clinical OI.^13^^,^^15^ While no clear etiology of this phenomenon has yet been described, we believe that this is likely due to the fact those with *COL1A1/1A2* variants have structural collagen defects whereas those with non-*COL1A1/1A2* variants usually have defects in the post-translational modification of bone and collagen.

We also found that individuals with *COL1A1/1A2* variants were significantly more likely (*p* = 0.0009) to have co-existing DI compared to those with non-*COL1A1/1A2* variants. While many OI genotype–phenotype studies have examined the relationship to DI, a clear relationship has not been defined. Some studies have shown that individuals with qualitative collagen defects are more likely to have DI,^13^ and others have found no difference in those with autosomal dominant inheritance when compared to those with autosomal recessive inheritance.^13^^,^^16^

Individuals with *COL1A1/1A2* variants were significantly more likely (*p* = 0.03) to have a documented family history of OI compared to those with non-*COL1A1/1A2* variants. This is consistent with the current understanding that those with *COL1A1/1A2* variants are inherited in an autosomal dominant nature, and those with non-*COL1A1/1A2* are inherited in an autosomal recessive nature.^19^

The significant difference in blue sclera, DI, family history of OI between patients with *COL1A1/1A2* and non-*COL1A1/1A2* variants can all inform the diagnosis, treatment, and management of OI patients based on their genetic variants. Blue sclera, DI, and family history are significantly more associated with having a *COL1A1/1A2* variant than a non-*COL1A1/1A2* variant. DI being more significant in *COL1A1/1A2* shows a potential greater need for tailored dental care for *COL1A1/1A2* patients. Blue sclera and DI can be visually perceptible hallmark signs of OI to primary care providers trying to distinguish between possible diagnoses and who may not encounter many OI patients regularly in their practice but are not definitive for making an OI diagnosis.

While this may suggest that there would be delayed treatment of patients with non*-COL1A1/1A2* variants due to a significant difference in some of the more unique visual signs commonly attributed to OI, there was no significant difference in if patients had ever received a DEXA scan, the age of the patient during their first DEXA scan, or the Z-score from their DEXA scans suggesting no substantial delay in care is evident. Similarly, there was no significant difference in whether bisphosphonate therapy was used or age at first infusion.

There was a significant difference between the *COL1A1/1A2* and non-*COL1A1/1A2* groups regarding history of scoliosis, but no significant difference between the 2 groups on if they had received scoliosis surgery. This finding might suggest a benefit in more frequent or proactive screening for scoliosis for patients with non-*COL1A1/1A2* variants for tracking scoliosis development, given that there is currently no standard of care regarding X-ray surveillance for scoliosis in patients with OI. Along the same line, there is no statistically significant difference between *COL1A1/1A2* and non-*COL1A1/1A2* groups and history of rod placement for long bone deformities. This would suggest that both groups should have the same screening and physical examination standards for assessing candidacy for rod placement surgery.

There was no significant difference between groups regarding the presence of hearing loss or the age at which they were first diagnosed with hearing loss, suggesting that similar screening practices should be maintained for both *COL1A1/1A2* and non-*COL1A1/1A2* patients. There was a significant difference however, in the development of expressive language disorder/delay with higher prevalence in the non-*COL1A1/1A2* group. During annual OI standard of care visits, any suspected expressive language delay or disorders should be referred to a speech language pathologist for early intervention, with no considerations for variant type. Making providers aware that expressive language disorder/delay is more prevalent in patients with non-*COL1A1/1A2* variants can be used to raise awareness and ensure timely intervention. There is no significant difference between *COL1A1/1A2* groups and non-*COL1A1/1A2* groups on the diagnosis of ASD. Similarly, any suspected ASD should be referred to the appropriate care provider for diagnosis and management.

There were few recorded instances of OI patients experiencing seizures, tics, or VPS in either group. It was initially thought that non-*COL1A1/1A2* patients might have more neurological manifestations than *COL1A1/1A2* patients since different genes are implicated in pathogenesis,^20–23^ but no difference was noted in the present analysis.

A patient's joint hypermobility and short stature can impact PT or OT recommendations or accommodations. The majority of both *COL1A1/1A2* and non-*COL1A1/1A2* patients have joint hypermobility, with no statistical difference between the 2 groups in prevalence. Similarly, there was no difference between the 2 groups in regard to being diagnosed with short stature. There being no difference between the 2 groups for both joint hypermobility and short stature would suggest no need to develop specified screening or management plans for patients belonging to either the *COL1A1/1A2* or non-*COL1A1/1A2* groups. Growth curves for *COL1A1/1A2-*related type III and IV OI already exist^24^; further evaluation of application of these curves for individuals with other genetic causes of severe and progressive OI are indicated.

While this cross-sectional study was still limited in its predictive power, comparing autosomal dominant and autosomal recessive forms of the disease due to a low number of patients in the non-*COL1A1/1A2* group, it supported the prevalence of underlying genetic causes of OI with roughly 90% (*n* = 268) of our patients carrying *COL1A1/1A2* variants which is consistent with well-established incidence data.^1^ As with other studies, the relatively low number of individuals with non-*COL1A1/1A2* related OI limited the predictive power of this study. Furthermore, the lack of sex differences between the *COL1A1/1A2* and non-*COL1A1/1A2* suggests an even distribution of variants among males and females. In conclusion, this genotype/phenotype analysis in pediatric patients with OI shows similar distribution between the COL and non-COL patients. The increased incidence of expressive language disorder/delay is a particularly interesting finding and may warrant further exploration of concomitant genetic disorders or nearby genes that could contribute to this finding.

## Data Availability

The data underlying this article cannot be shared publicly due to the sensitive nature of the data. The data will be shared on reasonable written request to the corresponding author.
